# The Bender-Gestalt Test: A Systematic Review

**DOI:** 10.7759/cureus.81122

**Published:** 2025-03-24

**Authors:** Hicham Lafhal, Ahami Omar Tohami Ahami, Kawtar Chafik, Siham Goutou, Rochdi Atmane

**Affiliations:** 1 Biology, Faculty of Sciences, Ibn Tofail University, Kenitra, MAR; 2 Laboratory of Natural Resources and Sustainable Development, Faculty of Sciences, Ibn Tofail University, Kenitra, MAR; 3 Rehabilitation, Higher Institute of Nursing Professions and Health Techniques (ISPITS) Rabat, Ministry of Health and Social Protection, Rabat, MAR; 4 Laboratory of Health Sciences and Techniques, Higher Institute of Health Sciences, Hassan I First University of Settat, Settat, MAR; 5 Health Sciences, Higher Institute of Nursing Professions and Health Techniques (ISPITS) Rabat, Ministry of Health and Social Protection, Rabat, MAR; 6 Continuing Education, Ibn Sina University Hospital Center, Rabat, MAR

**Keywords:** bender-gestalt test, interpretation, neuropsychology, scoring, validation, visuomotor assessment, visuomotor function

## Abstract

The Bender-Gestalt test (Bender test), since its appearance, has been widely used to analyze visuomotor functions, perception and cognitive processes. However, it is often used in a variety of other contexts. The aim of this research is to study the use of the Bender test and the Bender-II, based on the results of studies conducted between 2013 and 2023.

The search was carried out using inclusion and exclusion criteria and in accordance with the Preferred Reporting Items for Systematic Reviews and Meta-Analyses. The databases consulted were Medline, Embase and SciELO (Scientific Electronic Library Online), with no language restrictions. Study selection criteria included three steps: title, abstract and full text. The heterogeneity of study results precluded meta-analysis.

Sixty-two articles were retrieved through the electronic search, and after reviewing abstracts and reading full texts, 25 articles were considered to meet our inclusion criteria. Among these, studies reveal that the Bender test is particularly useful for diagnosing neurodegenerative disorders such as dementia and Alzheimer's disease. In particular, several studies have demonstrated its effectiveness in assessing visuomotor functions and visual perception, with mixed results regarding its sensitivity and specificity. Key findings include improved detection of cognitive impairment in the early stages of Alzheimer's disease and a strong association between test performance and patients' neuropsychological status.

In conclusion, although the Bender test is widely recognized and used in various clinical populations, its use remains subject to certain limitations. The main criticism concerns subjectivity in interpreting results and the variability of scores depending on cultural factors and the age of the individual.

## Introduction and background

The visuomotor Gestalt test, better known as the Bender-Gestalt test (Bender test), has been one of the most widely used psychological assessment instruments for over half a century. Since its first publication in 1938, this test has remained a benchmark for clinicians, schoolchildren, forensic scientists and neuropsychologists, thanks to its simplicity of application and its effectiveness in assessing visuo-motor abilities. Beyond its role in cognitive and developmental assessment, the Bender test has also been used to measure personality and emotional state [1].

The Bender test is based on assessing the precision and coordination of motor responses during the manual reproduction of nine geometric figures on a blank sheet of paper. Koppitz (1964) introduced a standardized scoring system that objectifies the assessment by assigning points to errors made when copying the figures [2]. This system has shown that the test score decreases with age until it stabilizes at around zero at 12 years of age, testifying to progressive and predictable visuomotor development in children.

The geometric shapes of Bender's test are taken from a selection of 30 figures developed by Wertheimer (1923), designed to illustrate the principles of Gestalt perception. Indeed, Wertheimer emphasized the ability of individuals to respond coherently to visual stimuli, and Bender followed up his work by demonstrating that perceptual-motor delays or organic and functional pathologies could lead to abnormal test performance [3].

Despite the widespread clinical use of the Bender test, this original version has been the subject of much criticism regarding its format and norms. Indeed, researchers have pointed to the absence of a standardized format, the lack of normative data and sometimes weak construct validity, raising questions about the reliability of the results obtained. These limitations led to the development of a revised version, the Bender-II, which introduced substantial modifications to the structure and functions of this traditional test, which has been the subject of several independent publications in Anglo-Saxon literature.

This systematic review aims to synthesize research conducted between 2013 and 2023 on the Bender ​​​​​​test and the Bender-II, highlighting their clinical applications, limitations and future research prospects.

## Review

Materials and methods

This systematic review followed the Preferred Reporting Items for Systematic Reviews and Meta-Analyses (PRISMA) guidelines to ensure the transparency and methodological rigor of the study.

Search Strategy

To include randomized controlled studies, observational studies, as well as reviews, systematic reviews, and meta-analyses related to the Bender test, the Medline (PubMed), Embase, and SciELO (Scientific Electronic Library Online) databases were searched in October 2023. The search was conducted using the Medical Subject Headings (MeSH) search, with terms such as "Bender Visual-Motor Gestalt Test" [MeSH], "Bender Test" [MeSH], "Bender Gestalt Visuomotor Test" [MeSH], and "Visual-Motor Performance Test". These terms were combined with the Boolean operators AND, OR, and NOT to refine the results.

Selection Criteria

This is a systematic review in which we included qualitative and quantitative studies that met our inclusion criteria. We considered articles published over the last decade, between 2013 and 2023, and without language restriction. All articles included in this review addressed the role of the Bender test in visuomotor and psychometric assessment in various contexts, such as neurological disorders, cognitive deficits, child neuropsychology and developmental disorders. In addition, we excluded studies and information published only in abstract form.

Due to variability in sample age, different techniques and treatments among study participants, and the discovery of non-evaluable articles, a meta-analysis was not performed.

Valuation Methods

The included studies were evaluated to assess the quality of the current literature using specific assessment tools: for clinical studies, the Jadad Scale for Randomized Controlled Trials [4]; for observational studies, the Newcastle-Ottawa Scale (NOS) [5]; and for reviews, the A MeaSurement Tool to Assess Systematic Reviews 2 (AMSTAR 2) scale [6]. A data extraction table was used to identify data relevant to the study, such as author name, year, study title, objectives, study method, groups compared, sample, consideration of confounding and bias, validity of tools and conclusions supported by results.

Results

**Figure 1:**
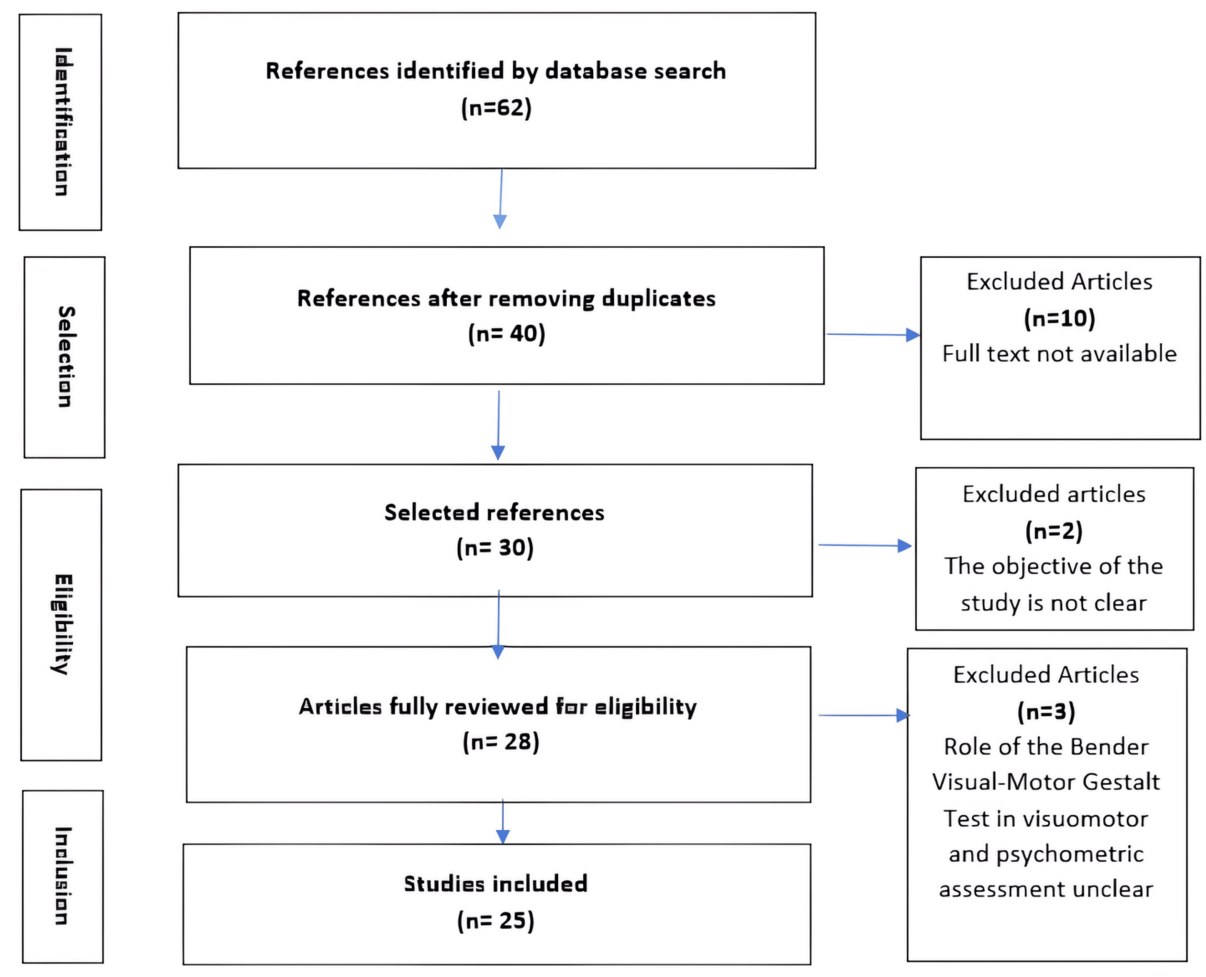
Flowchart of the study and results selection process

The results of our study showed a paucity of literature addressing the role of the Bender test in visuomotor and psychometric assessment in various near-miss neonatal contexts over the last few decades, between 2013 and 2023. In fact, only 62 articles were retrieved by electronic search, and after reviewing the various abstracts and reading the full texts, 25 articles were deemed eligible, meeting our inclusion criteria (Figure 1). The quality assessment of the selected studies is presented in Table 1.

**Table 1:** Evaluation of studies included in the review

Author, year	Type of study	Relevance to this study	Clearly stated objectives	Appropriate study method	Sample	Consideration of confounding factors and biases	Validation of questions	Tables/understandable figures	Conclusions supported by the results
Korkmaz et al., 2023 [7]	Psychometric study	Yes	Yes	Yes	Yes	No	Yes	Yes	Yes
Mufti et al., 2021 [8]	Comparative cross-sectional study	Yes	Yes	Yes	Yes	Yes	Yes	Yes	Yes
Keppeke et al., 2022 [9]	Correlational study	Yes	Yes	Yes	Yes	Yes	Yes	Yes	Yes
Keppeke et al., 2018 [10]	Correlational study	Yes	Yes	Yes	Yes	Yes	Yes	Yes	Yes
Mosotho et al., 2017 [11]	Retrospective exploratory study	Yes	Yes	Yes	Yes	Yes	Yes	Yes	Yes
Gulleroglu et al., 2013 [12]	Prospective study	Yes	Yes	Yes	Yes	Yes	Yes	Yes	Yes
Dos Santos et al., 2014 [13]	Transcultural study	Yes	Yes	Yes	Yes	No	Yes	Yes	Yes
Chan et al., 2019 [14]	Trial controll ed trial	Yes	Yes	Yes	Yes	Yes	Yes	Yes	Yes
Chellappa et al., 2018 [15]	Cross-over experimental study randomized	Yes	Yes	Yes	Yes	Yes	Yes	Yes	Yes
Kausar et al., 2021 [16]	Comparative study	Yes	Yes	Yes	Yes	Yes	Yes	Yes	Yes
Haritha et al., 2024 [17]	Observational study	Yes	Yes	Yes	Yes	Yes	Yes	Yes	Yes
Done et al., 2016 [18]	In-vivo study	Oui	Oui	Oui	Oui	Non	Oui	Oui	Oui
Samuel, 2020 [19]	Pilot study	Yes	Yes	Yes	Yes	Yes	Yes	Yes	Yes
Mukherjee et al., 2018 [20]	Case study	Yes	Yes	Yes	Yes	No	Yes	Yes	Yes
Kılıç et al., 2020 [21]	Pilot study	Yes	Yes	Yes	Yes	Yes	Yes	Yes	Yes
Cunha et al., 2019 [22]	Observational study	Yes	Yes	Yes	Yes	Yes	Yes	Yes	Yes
Cecato et al., 2020 [23]	Cross-sectional study	Yes	Yes	Yes	Yes	Yes	Yes	Yes	Yes
Blazkova et al., 2020 [24]	Cross-sectional study	Yes	Yes	Yes	Yes	Yes	Yes	Yes	Yes
Karami et al., 2019 [25]	Comparative study	Yes	Yes	Yes	Yes	Yes	Yes	Yes	Yes
Thurston et al., 2022 [26]	Longitudinal study	Yes	Yes	Yes	Yes	Yes	Yes	Yes	Yes
Kar et al., 2023 [27]	Cross-sectional study	Yes	Yes	Yes	Yes	Yes	Yes	Yes	Yes
Blazkova et al., 2022 [28]	Cross-sectional study	Yes	Yes	Yes	Yes	Yes	Yes	Yes	Yes
Jaszke-Psonka et al., 2016 [29]	Cohort study	Yes	Yes	Yes	Yes	Yes	Yes	Yes	Yes
Pandey et al., 2022 [30]	Observational study	Yes	Yes	Yes	Yes	Yes	Yes	Yes	Yes
Boostani et al., 2017 [31]	Experimental study	Yes	Yes	Yes	Yes	Yes	Yes	Yes	Yes

The studies gathered in this systematic review used various versions of the Bender test, including visuomotor and psychometric assessments in several contexts, such as neurological disorders, cognitive deficits, child neuropsychology and developmental disorders (Table 2).

**Table 2:** Comparison of characteristics and results of studies included in the review BGT: Bender Visual Motor Gestalt Test; BGT II: Bender Gestalt Test II; GISD B: General Intelligence and Short-Term Memory Diagnostic Battery; RCPM: Raven Colored Progressive Matrices; SPSS: Statistical Package for the Social Sciences; TNF alpha: Tumor necrosis factor alpha; ASA: American Society of Anesthesiologists classification; MMSE: Mini-Mental State Examination; BVRT: Benton Visual Retention Test; DKA: Diabetic ketoacidosis; SCA: Sudden cardiac arrest; PM2.5: Particulate matter with a diameter of fewer than 2.5 micrometers; N2O: Nitrous oxide

Author, year	Type of study	Rating scale	Scale score	Aim of the study	Study population	Parameters analyzed	Assessment methods
Korkmaz et al., 2023 [7]	Psychometric study	Bender-Gestalt II (BGT II) test	Not specified	Standardize the BGT II test and evaluate its psychometric properties on a Turkish sample, while obtaining normative values.	2691 Turkish students aged 4.0 to 17/month from various socio-economic backgrounds (low, medium, high)	Absolute agreement values for copy items, test-retest reliability coefficients for copy scores	Developmental Schedules, Colored Progressive Matrices, non-verbal intelligence test
Mufti et al., 2021 [8]	Comparative cross-sectional study	BGT II test	Not specified	Study neuropsychological functioning in children with and without specific learning disabilities (SLD).	116 children in Pakistan (64 with SLD and 52 without)	Memory function, with a comparison of recall scores between the two groups	BGT II (copy, recall, perceptual, and motor test subscales), SPSS 21 analysis
Keppeke et al., 2022 [9]	Correlational study	Bender Visual-Motor Gestalt Test (BGT)	Not specified	Examine the correlation between visual-motor development (as measured by the BGT) and sexual maturity, according to Tanner stages.	134 adolescents aged 10 to 15	Visual-motor development, sexual maturity (Tanner stages), Raven scores, effects of grade repetition and developmental disorders	BGT, Raven's Progressive Matrices Test, information on sexual maturity and developmental disorders from medical records
Keppeke et al., 2018 [10]	Correlational study	BGT, Raven's Progressive Matrices Test	Not specified	Study the relationship between visual-motor development and pubertal changes using the Tanner scale.	134 teenagers aged 10 to 15	Visual-motor development and pubertal changes (Tanner stages), Raven scores, influence of schooling, repetition, and developmental problems	BGT, Raven's Progressive Matrices Test, medical data on pubertal maturity
Mosotho et al., 2017 [11]	Retrospective exploratory study	BGT, CT scan	Not specified	Examine the association between BGT results, CT scans and assessment of competency to stand trial and criminal responsibility.	Patient population at the Free State Psychiatric Complex (FSPC), South Africa, 2013	Association between BGT scores (Hain) and outcomes of criminal responsibility and competency to stand trial, presence of brain damage	BGT, CT scan, demographic and clinical data from medical records
Gulleroglu et al., 2013 [12]	Prospective study	BGT, Cancellation Test, Visual and Auditory Number Assay Test	Not specified	Determining neurocognitive function in pediatric kidney transplant patients.	20 kidney transplanted children (aged 6-16), with control groups of 20 healthy children and 20 with chronic renal failure	Neurocognitive functions: frequent dysfunction after transplantation	BGT, Cancellation Test, Visual and Auditory Number Assay Test
Dos Santos et al., 2014 [13]	Transcultural study	Bender-Gradual Scoring System (B-SPG)	Not specified	Examine the psychometric qualities of the B-SPG system for assessing perceptual-motor maturity independently of cultural context.	231 children: three Brazilian states, 231 children aged 6 to 10, including 108 from various regions of Lima (Peru) and 123 from three Brazilian states	Perceptual-motor maturity and the influence of cultural context	Group projection of figures, correction by experienced psychologists following instructions in Portuguese and Spanish
Chan et al., 2019 [14]	Randomized controlled trial	BGT, Hand-eye coordination test, Cognitive function test	Not specified	Investigating the effects of interactive cognitive-motor training on hand-eye coordination and cognitive function in the elderly.	62 elderly people, divided into experimental and control groups	Hand-eye coordination, motor sub-capacities, visual perception, cognitive function	Immediate, three-month and six-month post-test assessments
Chellappa et al., 2018 [15]	Randomized experimental crossover study	Sustained attention, cognitive output, information processing, visual-motor performance	Not specified	Investigating the impact of circadian misalignment on task-dependent human cognitive performance.	Individuals exposed to a night shift pattern	Sustained attention, cognitive output, visual-motor performance, sleep	Shift work paradigm with attention and performance measurement over several days
Kausar et al., 2021 [16]	Comparative study	BGT II	Not specified	Comparing cognitive impairment between patients with epileptic and psychogenic non-epileptic seizures.	125 patients, including 62 with epilepsy and 63 with psychogenic non-epileptic seizures	Cognitive disorders	Application of the BGT II to assess cognitive deficits
Haritha et al., 2024 [17]	Observational study	Stroop test, Wisconsin Card Sorting Test (WCST), Trail Making Test-B, BGT	Not specified	Compare the incidence of postoperative cognitive decline in elderly patients undergoing open abdominal surgery under general anesthesia with isoflurane or desflurane.	40 patients aged between 60 and 80	Postoperative cognitive decline, biomarkers (interleukin 1, interleukin 6, TNF alpha, amyloid β, S100)	Cognitive evaluation before and after surgery with tests and blood tests
Done et al., 2016 [18]	In-vivo study	BGT II	Not specified	Comparing the efficacy of oral sedation with midazolam-N2O and ketamine-N2O in children during dental treatment.	30 healthy children (3-9 years) (ASA I and II) requiring multiple tooth extractions	physiological parameters (respiratory rate, pulse rate, oxygen saturation), psychomotor performance	Physiological measurements (respiratory rate, pulse, oxygen saturation) and psychomotor test (modified BGT)
Samuel, 2020 [19]	Pilot study	BGT, WCST	Not specified	Investigating visuo-motor and executive functional deficits in adult patients with primary generalized epilepsy.	30 adults, including a target group of epilepsy patients and a healthy control group	Visual-motor and executive functioning	Visual-motor and executive functioning
Mukherjee et al., 2018 [20]	Case study	BGT, Binet Kamat Intelligence Test (BKT)	Average of 8.6 (BGT), average IQ of 85.5 (BKT)	Investigating the relationship between the location of mutations in the DMD gene and cognitive deficits in children with Duchenne muscular dystrophy.	10 male children aged 4 to 9 with genetic mutations	Visual-motor function, IQ, effects of mutation localization	BGT, BKT
Kılıç et al., 2020 [21]	Pilot study	Wechsler Intelligence Scale for Children-Revised (WISC-R), BGT	Specific subtest results: p=0.024 (image completion), p=0.001 (image arrangement)	Examining neuropsychological development in children with primary monosymptomatic nocturnal enuresis.	30 children with nocturnal enuresis, 30 healthy children	Neuropsychological development, cognitive tests, visual-motor functioning	WISC-R, BGT
Cunha et al., 2019 [22]	Observational study	WISC, BGT, Auditory Processing Evaluations (APE)	APE tests and lower parental education	Study the cognitive profiles of children with SLD, with an assessment of auditory processing.	34 children with LTC and 15 control children (ages 7-14)	Auditory processing, verbal and spatial reasoning, cognitive performance	Hearing tests (tone audiometry, acoustic immittance, brainstem evoked response), Wechsler Scale tests, BGT
Cecato et al., 2020 [23]	Cross-sectional study	BGT	3.2 (healthy control group), 7.21 (AD patients), 8.04 (VD patients), p<0.0001	Evaluating the performance of elderly people on the BGT and using error types to discriminate between healthy control groups, Alzheimer's patients and vascular dementia.	285 elderly people (over 60, >1 year of schooling), divided into control, Alzheimer's and vascular dementia groups	BGT score differences between groups, types of execution errors	Clinical history, neuropsychological tests (BGT, CAMCOG MMSE, GDS, PFAQ), neuroimaging
Blazkova et al., 2020 [24]	Cross-sectional study	BGT, Raven Colored Progressive Matrices (RCPM)	No significant difference observed between the groups	Analyzing the impact of polycyclic aromatic hydrocarbons (PAHs) on the cognitive development of five-year-old children.	169 five-year-old children from Karvina (n = 70) and Ceske Budejovice (n = 99)	PAH exposure during pregnancy and cognitive development in children (BGT, RCPM)	B[a]P and OH-PAH concentrations in air and urine, psychological tests (BGT, RCPM)
Karami et al., 2019 [25]	Comparative study	BGT II	Epilepsy patients scored lower than healthy controls and migraine patients lower than healthy controls	Comparing cognitive and perceptual functions in patients with occipital epilepsy, migraine patients and healthy individuals.	93 participants (epilepsy patients, migraine sufferers and healthy controls)	Group differences (occipital epilepsy, migraine, healthy controls) on copying, recall, motor and perceptual subscales	Demographic questionnaire, BGT II
Thurston et al., 2022 [26]	Longitudinal study	Statistical links BGT, WCST	Statistical links between postnatal methylmercury exposure and neurodevelopmental outcomes at 9, 17, 22, and 24 years of age	Study the effects of postnatal methylmercury exposure on neurodevelopmental outcomes over a 24-year period.	312-550 participants (Seychelles Child Development Study main cohort)	Associations between methylmercury exposure (via hair measurements) and 85 neurodevelopmental outcomes at different ages (9, 17, 22, and 24 years)	Measurements of methylmercury in hair at different ages, adjusted linear regressions
Kar et al., 2023 [27]	Cross-sectional study	WISC-R, GISD-B, BGT	Poorer cognitive function in children with poor metabolic control and a history of DKA	Examine the effects of age of diabetes onset, metabolic control and type of insulin regimen on neurocognitive function in children and adolescents with type 1 diabetes.	47 children (aged 6-18), type 1 diabetes for at least five years	Intelligence (WISC-R), short-term memory (GISD-B), visual-motor perception (BGT), attention (Moxo-dCPT), hyperactivity, impulsivity	Measurement of specific neurocognitive tests (WISC-R, GISD-B, BGT, Moxo-dCPT)
Blazkova et al., 2022 [28]	Cross-sectional study	BGT, RCPM	Links between prenatal oxidation and cognitive performance	Studying the impact of oxidative damage linked to PM2.5 particles during the prenatal period on the cognitive development of children at five years of age.	Five-year-old children, n = 169, born in 2013 and 2014, living in Karvina (polluted area) and Ceske Budejovice (control area)	Prenatal PM2.5 levels, oxidation biomarkers in urine and plasma (8- oxodG, 15-F2t-IsoP), cognitive tests	Measurement of PM2.5 levels during 3rd trimester of pregnancy, cognitive tests (BGT and RCPM)
Jaszke-Psonka et al., 2016 [29]	Cohort study	MMSE, Digit Span Test, BGT, BVRT	Disparities in cognitive function between study and control groups	To assess the incidence and severity of cognitive deficits in survivors of SCA compared with those who suffered a myocardial infarction without SCA and a healthy control group.	Cardiac arrest survivors (n = 30), MI survivors without ACS (n = 31), healthy control group (n = 30)	Incidence of cognitive deficits, duration of cardiac arrest	Cognitive tests (MMSE, Digit Span, BGT, BVRT)
Pandey et al., 2022 [30]	Observational study	Pulse oximeter, BGT, Electric pulp tester	Physiological, psychomotor and analgesic changes	Assessing variations in physiological, psychomotor and analgesic parameters during nitrous oxide (N2O) titration in children aged 3 to 12 years.	100 children aged 3 to 12	Oxygen saturation, heart rate, pain and psychomotor performance	Oximeter, BGT, electrical pulpometer test
Boostani et al., 2017 [31]	Experimental study	BGT (digitized)	Classification accuracy	Developing a quantitative method for diagnosing obsessive-compulsive disorder (OCD) using the drawing characteristics of the BGT.	50 OCD patients under treatment, 50 control subjects	Drawing characteristics such as pen angle, speed, curvature and pressure	Optical pen, digital tablet, Hidden Markov Model (HMM), Multilayer Perceptron Model (MLP)

Discussion

The results of this systematic review of studies on the use of the Bender test highlight its wide application in various fields, including neuropsychology, psychiatry and cognitive disorders, as well as its growing role in cognitive aging research. This tool remains central to the assessment of cognitive and neuropsychological disorders, particularly after traumatic events such as stroke or head injury [32].

In addition to its use in cognitive disorders, the Bender test also finds applications in the assessment of developmental disorders, such as autism spectrum disorder (ASD) and attention deficit hyperactivity disorder (ADHD). Studies show that the performance of children suffering from these disorders is often impaired on the Bender test, particularly in areas such as motor planning and visual memory. However, some researchers point out that this test, while effective in some areas of cognitive assessment, does not capture the full complexity of developmental disorders, particularly those related to social and behavioral aspects. In addition, the Bender test plays a key role in the study of cognitive aging, particularly in the context of neurodegenerative disorders such as Alzheimer's disease [23].

Despite its many applications, the Bender test has certain limitations that can affect the accuracy of the assessment. It seems less sensitive for detecting mild cognitive deficits, particularly in the early stages of cognitive impairment. In addition, external factors such as stress, fatigue or the patient's emotional state at the time of assessment may influence performance, raising concerns about the validity of the results. These factors underline the importance of a holistic assessment and contextual interpretation of Bender test results, in order to compensate for its limitations and maximize its relevance in clinical practice [33].

Strengths and Implications of the Study

The study has several strengths, including its adherence to the PRISMA 2020 guidelines, which ensures methodological rigor and transparency in the research process. It provides a comprehensive analysis of the Bender test in various clinical settings, including neurological disorders and cognitive deficits. A major strength lies in the use of rigorous quality assessment tools, ensuring the reliability of the included studies. However, the review highlights the paucity of recent publications on the topic, which represents both a limitation and an opportunity for future research. The practical implications of this review highlight the need for better standardization of assessment practices and increased exploration of the Bender test in lesser-studied populations. This paves the way for future studies that could fill these gaps and refine the application of this test in diverse settings.

## Conclusions

The Bender test remains a fundamental tool in neuropsychological assessment, providing crucial information on patients' visuospatial abilities and cognitive perception. Its use is well documented in a number of clinical fields, including the assessment of neurocognitive disorders, developmental disorders and cognitive aging. It has demonstrated its effectiveness in the diagnosis of neurological and psychiatric disorders, such as after a stroke or head injury, and in pathologies such as ASD and ADHD. However, its sensitivity to mild cognitive disorders and variability influenced by socio-cultural and educational factors necessitate cautious and often complementary use with other diagnostic tools.

The growing use of the Bender test in the context of cognitive aging and neurodegenerative disorders, particularly Alzheimer's disease, shows its potential for the early detection of these pathologies. However, to maximize its effectiveness, it needs to be integrated into a broader neuropsychological test battery. Future research should focus on adapting the test to specific populations and improving its cultural validity, in order to optimize its usefulness in monitoring cognitive disorders over time.
